# Toll-like receptors, innate immune system, and lung diseases: a vital trilateral association

**DOI:** 10.17179/excli2022-4688

**Published:** 2022-02-25

**Authors:** Vyoma K. Patel, Keshav R. Paudel, Shakti D. Shukla, Gang Liu, Brian G. Oliver, Philip M. Hansbro, Kamal Dua

**Affiliations:** 1 Macular Disease Foundation, NSW 2000, Australia; 2 Faculty of Health, Australian Research Centre in Complementary and Integrative Medicine, University of Technology Sydney, Ultimo, NSW 2007, Australia; 3 School of Life Sciences, University of Technology Sydney, Sydney, NSW 2007, Australia; 4 Centre for Inflammation, Centenary Institute, Sydney, NSW 2050, Australia; 5 Discipline of Pharmacy, Graduate School of Health, University of Technology Sydney, Ultimo, NSW 2007, Australia; 6 Woolcock Institute of Medical Research, University of Sydney, Sydney, New South Wales, Australia

## ⁯

The lung is highly susceptible to external environmental pollutants such as infectious agents and foreign antigens and thus, expresses an extensive repertoire of pathogen recognition receptors (PRRs) to recognize both exogenous pathogen-associated and endogenous danger-associated molecular patterns (Jiang et al., 2006[9], Lipinski et al., 2021[12]). Notably, Toll-like receptors (TLR) play a key role in this process as they are located on cell surface as well as intracellularly (Lipinski et al., 2021[12]; Maris et al., 2006[13]). 

TLR2 and TLR4 are the key players in the onset of asthma including the inflammatory responses underlying asthmatic exacerbations as they are present in both the airway epithelium and macrophages, and the binding of these products results in allergic inflammation (Millien et al., 2013[14]). Notably, an exposure to allergens such as house dust mites (HDM) leads to the activation of TLR2 and TLR4 and subsequent Th2 cells that are allergen-specific that further lead to imbalance in Th1/Th2 responses (Lafferty et al., 2010[10]; Phipps et al., 2007[19]). Interestingly, TLR signaling is regulated by two distinct pathways such as myeloid differentiation factor 88 (MyD88)-dependent and MyD88-independent. The interaction between MyD88 and Toll-like receptors further flare-up inflammatory response leading to the release of cytokines such as TNF-α, IL-1β, CXCL10, IL-6, and IFN-γ (Piras and Selvarajoo; 2014[20]). In addition, studies have shown the increased expressions of TLR2, TLR3, and TLR4 in patients with severe asthma as well as associated morbidity, suggesting their potential role in the development of severe or even fatal asthmatic exacerbations (Lipinski et al., 2021[12]; Ferreira et al., 2012[5]; Hansbro et al., 2017[8]).

Interestingly, TLRs (such as TLR2, TLR4, and TLR9), also contribute to the pathogenesis of COPD (Hansbro et al., 2017[8]). Studies have shown that the exposure to cigarette smoke (one of the major instigator of COPD) significantly correlate with the increased gene expression of both Toll-like receptors, TLR4 and TLR9 as well as cytokine production (Bezemer et al., 2012[2]; Nadigel et al., 2011[16]). Moreover, this increased gene expression of TLR4 further leads to upregulation of IL-8 from T cells and associated neutrophil recruitment (Nadigel et al., 2011[16]; Mortaz et al., 2010[15]). Studies have shown that alveolar apoptosis is increased in TLR4-deficient mice upon cigarette smoke exposure, thus suggesting a protective role of TLR4 in cellular regulation and prevention of apoptosis (An et al., 2012[1]). Indeed, studies have shown that mice that lack TLR4 expression are more susceptible to oxidative stress, and this is mainly due to an upregulation of nicotinamide adenine dinucleotide phosphate (NADPH) oxidase - a regulator of reactive oxygen species (ROS) (Zhang et al., 2011[30]). Thus, these findings suggest that in dearth of TLR4, the upregulation of ROS may contribute to cellular apoptosis and emphysema, highlighting the key role of Toll-like receptors in COPD.

The initial research into COVID-19, the ongoing devastating pandemic, has revealed the involvement of TLRs, especially TLR2 and MyD88, that were critical for β-coronavirus-induced inflammatory responses (through sensing protein E of coronaviruses). Moreover, TLR2-dependent signaling resulted in heightened production of proinflammatory cytokines during coronavirus infection, which was independent of viral entry, and led to the 'cytokine storm'. Furthermore, blockade of TLR2 resulted in mitigation of SARS-CoV2 infection (Zheng et al., 2021[31]). Another study suggests that younger males with TLR7loss-of-function variant of the gene exhibit a more severe form of COVID when compared to age-matched males who had a normal gene variant (Fallerini et al., 2021[4]). Importantly, TLR7 has been implicated in recognition of ssRNA viruses, including SARS-CoV2 (Poulas et al., 2020[21]). 

Generally, the influenza virus is recognized by TLR7 that binds ssRNA and TLR3, which senses dsRNA in the endosomes (Poux et al., 2019[22]). However, novel roles of other TLRs are being constantly investigated and reported. For instance, a study has recently reported that activating TLR2 in nasal epithelial cells (via a novel TLR 2 activating compound), usually the first site of respiratory viral infections, could very well mitigate influenza (A) viral infection and associated pathology in the lower lung (Deliyannis et al., 2021[3]). This becomes especially relevant to vulnerable populations that demonstrate a poor response to conventional vaccines and/or therapies (Deliyannis et al., 2021[3]). This also highlights the importance of novel TLR-based therapies that provide clinically relevant anti-viral effects. Another TLR, TLR10, often referred to as an 'orphan' receptor has been shown to be crucial in induction of pro-inflammatory cytokines and interferons in primary human peripheral blood monocyte-derived macrophages and human monocytic cell line treated with influenza virus. Moreover, the magnitude of immune response activation depended upon the virulence of the influenza virus utilized in the experiments (Lee et al., 2014[11]). 

Two TLRs, TLR2 and TLR4, have been implicated in recognition and clearance of *Haemophilus influenzae* from the lung. For instance, mice expressing TLR4 showed rapid clearance of *H. influenzae* when compared to mice deficient in TLR4. The bacterial clearance was aided by induction of pro-inflammatory cytokines, including IL-1β, IL-6, TNF-α, macrophage-inflammatory protein (MIP)-1α, and MIP-2 in bronchoalveolar lavage, as well as recruitment of intrapulmonary neutrophils (Wang et al., 2002[29]). Recently, a study has reported a key role for TLR5-agonist (flagellin) in mounting protection against non-typeable *H. influenzae*-induced exacerbation in a mouse model of cigarette smoke-induced chronic obstructive pulmonary disease (Perez-Cruz et al., 2021[18]). The protection against exacerbation was associated with an early neutrophilia, a reduced production of pro-inflammatory cytokines, and an increased IL-22 production (Perez-Cruz et al., 2021[18]). Taken together, these studies highlight the therapeutic potential of TLR-based drugs in prevention/management of lung infections.

Considering the association of TLR in different inflammatory conditions including lung diseases, various TLR antagonists could be potential options for the management of these inflammatory disorders (Gao et al., 2017[6]). Inhibition of TLR is conceivable by two different approaches firstly, by blocking TLR ligands to bind with its receptor; and secondly, by interfering with the downstream cell signaling pathways to further inhibit the signal transduction. Various therapeutic categories such as oligodeoxynucleotides (ODNs), antibodies, lipid-A analogs, microRNAs (miRNA) are investigated for their potential to inhibit TLR signaling (Gao et al., 2017[6]). OPN-301 is an anti-TLR2-specific antibody that blocks TLR2 mediated cytokines such as TNF-α, IL-1β, IFN-γ and IL-8 production (Ultaigh et al., 2011[28]). Compound IMO-3100 is an oligonucleotide-based TLR7/9 dual antagonist that could notably inhibit the inflammatory gene expression including IL-17A, β-defensin, CXCL1, and keratin 16 in a mouse model (Suarez-Farinas et al., 2013[26]). Eritoran (E5564) is a synthetic lipid A analog design as TLR4 antagonist as it prevents lipopolysaccharide (LPS) binding and the induction of TLR4 signaling. In a pre-clinical animal model, it was observed that eritoran can remarkably decrease LPS-mediated NF-κB activation and pro-inflammatory cytokine (TNF-α, IL-1β, IL-6, and IL-8) production *in vitro *and in animal models (Savov et al., 2005[24]). Since LPS is known to cause airway inflammation both *in vitro *(Paudel et al., 2020[17]) and *in vivo *(Hadjigol et al., 2020[7]), these TLR antagonists could possibly prevent this disease. Among various potential miRNAs, miR-146a, miR-155, and miR-21 have been extensively studied for their regulatory roles in TLR signaling (Quinn and O'Neill, 2011[23]). miR-146 controls TLR and cytokine signaling through down-regulation of IL-1 receptor-associated kinase 1 and TNF receptor-associated factor 6 protein levels (Taganov et al., 2006[27]). miR-21 and miR-155 are found to be upregulated in TLR-associated inflammatory conditions such as macrophage-mediated inflammation and thus targeting these miRNAs could be another option to control TLR induced inflammation (Sheedy et al., 2010[25]). Further pre-clinical models and clinical studies investigating the role of TLR antagonists are essential to validate the use of TLR antagonists in respiratory diseases. Nevertheless, these various therapies could be beneficial to control airway inflammation as seen during the progression of acute and chronic respiratory disease. 

## Declaration

### Conflict of interest

The authors have no conflict of interests to declare. 

### Funding

None.
